# Antibacterial Therapy by Ag^+^ Ions Complexed with Titan Yellow/Congo Red and Albumin during Anticancer Therapy of Urinary Bladder Cancer

**DOI:** 10.3390/ijms23010026

**Published:** 2021-12-21

**Authors:** Anna Jagusiak, Tomasz Gosiewski, Dorota Romaniszyn, Małgorzata Lasota, Anna Wiśniewska, Katarzyna Chłopaś, Barbara Ostrowska, Izabela Kościk, Małgorzata Bulanda

**Affiliations:** 1 Chair of Medical Biochemistry, Faculty of Medicine, Jagiellonian University Medical College, 31-034 Kraków, Poland; malgorzata.lasota@uj.edu.pl (M.L.); barbara.ostrowska@uj.edu.pl (B.O.); izabela.koscik@uj.edu.pl (I.K.); 2 Chair of Microbiology, Department of Molecular Medical Microbiology, Faculty of Medicine, Jagiellonian University Medical College, 31-121 Krakow, Poland; tomasz.gosiewski@uj.edu.pl; 3 Chair of Microbiology, Department of Infection Control and Mycology, Faculty of Medicine, Jagiellonian University Medical College, 31-121 Krakow, Poland; d.romaniszyn@uj.edu.pl (D.R.); malgorzata.bulanda@uj.edu.pl (M.B.); 4 Chair of Pharmacology, Faculty of Medicine, Jagiellonian University Medical College, 31-531 Krakow, Poland; anna.niepsuj@uj.edu.pl; 5 Pulmonology and Allergology Clinical Department, University Hospital in Krakow, 30-688 Krakow, Poland; kchlopas@su.krakow.pl

**Keywords:** titan yellow, congo red, silver ions, bovine serum albumin, *Escherichia coli*, *Staphylococcus aureus*, minimum inhibitory concentration, minimum bactericidal concentration, T24 cell line, tumor growth, apoptosis, necrosis, migration

## Abstract

According to the World Health Organization report, the increasing antibiotic resistance of microorganisms is one of the biggest global health problems. The percentage of bacterial strains showing multidrug resistance (MDR) to commonly used antibiotics is growing rapidly. Therefore, the search for alternative solutions to antibiotic therapy has become critical to combat this phenomenon. It is especially important as frequent and recurring infections can cause cancer. One example of this phenomenon is urinary tract infections that can contribute to the development of human urinary bladder carcinoma. This tumor is one of the most common malignant neoplasms in humans. It occurs almost three times more often in men than in women, and in terms of the number of cases, it is the fifth malignant neoplasm after prostate, lung, colon, and stomach cancer. The risk of developing the disease increases with age. Despite the improvement of its treatment methods, the current outcome in the advanced stages of this tumor is not satisfactory. Hence, there is an urgent need to introduce innovative solutions that will prove effective even in the advanced stage of the disease. In our study, a nanosystem based on ionic silver (Ag^+^) bound to a carrier—Titan yellow (TY) was analyzed. The possibility of binding the thus formed TY-Ag system to Congo red (CR) and albumin (BSA) was determined. TY-Ag binding to CR provides for better nanosystem solubility and enables its targeted intracellular transport and binding to immune complexes. The binding of TY-Ag or CR-TY-Ag to albumin also protects the system against the uncontrolled release of silver ions. It will also allow the delivery of silver in a targeted manner directly to the desired site in the case of intravenous administration of such a system. In this study, the MIC (Minimum Inhibitory Concentration) and MBC (Minimum Bactericidal Concentration) values of the TY-Ag or BSA-TY-Ag systems were determined in two reference strains (*Escherichia coli* and *Staphylococcus aureus*). The paper presents nanosystems with a size of about 40–50 nm, with an intense antibacterial effect obtained at concentrations of 0.019 mM. We have also discovered that TY-Ag free or complexed with BSA (with a minimal Ag^+^ dose of 15–20 μM) inhibited cancer cells proliferation. TY-Ag complex diminished migration and effectively inhibited the T24 cell viability and induced apoptosis. On the basis of the obtained results, it has been shown that the presented systems may have anti-inflammatory and antitumor properties at the same time. TY-Ag or BSA-TY-Ag are new potential drugs and may become in future important therapeutic compounds in human urinary bladder carcinoma treatment and/or potent antimicrobial factors as an alternative to antibiotics.

## 1. Introduction

According to the World Health Organization report, the increasing antibiotic resistance of microorganisms is one of the biggest global health problems [1,2]. It is a very dangerous phenomenon because frequent and recurring infections can cause many neoplastic diseases, including human urinary bladder carcinoma.

The percentage of bacterial strains showing multidrug resistance (MDR) to commonly used antibiotics is growing rapidly. Therefore, there is an urgent need to develop new drugs to combat them [3,4,5]. In the last five years, most strains of the Gram-negative *Enterobacteriacae* species (such as *Klebsiella pneumoniae* and *Escherichia coli* (*E. coli*) recorded in southern and south-eastern Europe were resistant to third-generation cephalosporins, fluoroquinolones, and aminoglycosides [6]. Methicillin-resistant *Staphylococcus aureus* (MRSA) strains, once associated mainly with hospital-acquired MRSA (HA MRSA), are now also responsible for the majority of community-acquired staph infections (CA MRSA). Genetic studies have shown that the strains causing these infections often have the SCCmec type IV chromosome, which is not usually found in hospital MRSA strains. Thus, we are dealing with a new threat, and not with infectious hospital strains that got into the outside community [7]. One of the causes of this problem is the frequent use of antibiotics, which increased significantly between 2010 and 2020 in the European community. Unfortunately, the mere supervision of alarm strains, their registration, as well as the system controlling the consumption of antibiotics is insufficient in the fight against multidrug resistance of microorganisms. In the case of patients with urothelial carcinoma, the statistics show that the use of antibiotics contributes to a worse survival rate than in those who do not receive antibiotics [8]. 

Bladder cancer is diagnosed in more than 430,000 patients worldwide every year, making it the ninth most common malignancy. Referring to the data for the United States from 2020 on the *www.cancer.net* portal, it is almost three times more prevalent in men than in women [9]. Unfortunately, the etiology and pathophysiology of bladder cancer remain unknown. It may be caused by genetic mutations and external risk factors, including tobacco smoking, carcinogen exposure, the chlorination of drinking water, and possibly cyclophosphamide [10]. Bladder cancer most often (approx. 90%) originates from the cells of the transitional epithelium lining the bladder. Depending on the degree of differentiation of this neoplasm, the following forms can be distinguished: well-differentiated tumors with low malignancy potential (G1), medium-grade (G2) neoplasms, poorly differentiated, high-grade (G3) neoplasms, and undifferentiated, anaplastic neoplasms [11]. The disease may also develop as a consequence of previous radiotherapy or frequent and recurrent urinary tract infections. Transient inflammation is considered to be a part of the body’s immune defense against the pathogen, but persistent inflammation has the potential to contribute to the development of cancer of varying degrees of severity [12].

A long-term indwelling catheter use is also a risk factor for bladder cancer. Conversely, treatments for an already diagnosed bladder cancer are often associated with catheterization. Administration of drugs into the bladder also increases the risk of infection. Various types of intravesical chemotherapy are used. For example, thanks to the HIVEC system used immediately after the transurethral surgery to remove (resect) the tumor, the progression and recurrence of the disease are significantly reduced by about 2.5 times. Treatment is based on heating and recirculation of the chemotherapeutic between the apparatus and the inside of the bladder through the inserted catheter. Such action results in better penetration of the drug into cells, which results in greater effectiveness of the therapy. However, patients with HIVEC therapy exhibit symptoms of inflammation [13]. Finding a solution with the use of catheterization during recurrent urinary tract infections that will prevent the development of cancer seems to be an important research goal.

Therefore, the search for innovative solutions alternative to the commonly used antibiotic therapy has become a necessary requirement to combat the problem of microbial multidrug resistance, recurrent urinary tract infections, and urinary bladder carcinoma [14]. A promising alternative is the combination of chemotherapeutics with heavy metal ions, for example, silver. Unfortunately, silver in the ionic form from silver nitrate is very reactive. Moreover, in the presence of phosphates, chlorides, bromides, iodides, etc., silver can form sparingly soluble deposits. Hence, administering silver nitrate directly to the body is not safe and causes a condition called argyria. The administration of silver in the form of nanoparticles or complexation with a protective compound raises its reactivity threshold, especially in relation to -SH groups. Such silver complexing compounds include Titan yellow, which we have recently analyzed, and its combination with albumin. The reactivity of such complexed silver is increased (the dissociation constant of the complex is estimated at 10^−13^) because anions with Kd < 10^−13^ do not remove silver from the complex [15]. Thanks to this, a lower concentration of silver can be used, which significantly reduces its toxicity and additionally provides protection. Importantly, silver interacts with thiol groups that are present on the surface of many bacteria or are revealed on the surface of the bacteria during their division. Many authors argue that Ag^+^ ions interact with the functional groups of the proteins, e.g., thiol groups of proteins present in the cell membrane, resulting in their deactivation [16,17]. Both silver nanoparticles and Ag^+^ ions alter the 3D structure of proteins upon interaction, interfere with disulfide bonds, and block active binding sites leading to overall functional defects in the microorganism [18].

The present work compares the effect of free silver (AgNO_3_), silver complexed with TY (TY-Ag), its complex with albumin (BSA-TY-Ag), and the complexes with Congo red (CR) (CR-TY-Ag, BSA-CR-TY-Ag). CR is a supramolecular drug carrier, which additionally ensures a better solubility of the TY-Ag complex. Additionally, CR reduces silver toxicity and binds to antigen-bound antibodies. Therefore, research is underway on its use as a potential carrier factor for drugs, e.g., doxorubicin [19]. A new method of treating advanced bladder cancer, based on monoclonal antibodies (immunotherapy), the so-called inhibitors of immune checkpoints, has recently been available. It is an antibody against the PD-L1 protein present on the surface of urinary tract cancer cells intended, and FDA approved, as a second-line therapy in advanced bladder cancer [20,21]. Therefore, binding the CR-TY-Ag complex to antibodies against the PD-L1 protein can be an alternative treatment for urinary tract cancer.

The TY-Ag and CR-TY-Ag nano complexes have been partially described in [15]. On the other hand, the BSA-TY-Ag complexes have not been described in detail so far. Albumin increases the protection of the carried compounds against uncontrolled release, which should reduce their toxicity. 

Two reference strains were selected for the microbiological analyzes carried out in this study: *Escherichia coli* (*E. coli*; EC, ATCC^®^ 35218™) and *Staphylococcus aureus* (*S. aureus;* SA, ATCC^®^ 29213™). Free Ag^+^ ions show high antibacterial activity against Gram-positive and Gram-negative bacteria, but also against viruses and fungi [22]. The main bacterium isolated from cases of urinary tract infections (80%) was identified as *Escherichia coli,* although other bacteria were also cultured (*Klebsiella pneumoniae* or *Pseudomonas aeruginosa*) [23]. *S. aureus* may be a pathogen of the urinary tract among patients in long-term care facilities, wearing indwelling catheters, with urinary tract obstruction and/or malignant neoplasms, with instrumentation [24]. In these cases, an *S. aureus* bloodstream infection (SABSI) from an “ascending” urinary tract source (UTS) [25,26] may have developed. Hence, these two groups of bacteria were selected as model strains in this research.

## 2. Results

### 2.1. Characterization of TY-Ag Complexes with Protein (BSA-TY-Ag, BSA-CR-TY-Ag)

The characterization of the presented systems is the subject of this study. The size and stability of the resulting complexs were characterized by electrophoresis, gel filtration, UV-VIS spectrometry and DLS methods. The real effect of the added CR and BSA on the size of the systems and their effect on bacteria and neoplastic cells was assessed.

#### 2.1.1. TY-Ag Complexes with CR and/or BSA in the Optimal Molar Ratio of TY-Ag = 1:0.5 (Agarose Gel Electrophoresis)

The aim of the analysis was to show that ionic silver can bind to Titan yellow and then can form complexes with CR, which improves solubility (CR-TY-Ag), and with BSA, which provides a protective role. The binding efficiency of Ag^+^ to TY at different molar ratios of TY-Ag (1:1.6 and 1:0.8) was compared in order to optimize the complex for further use in microbiological analyzes. It has been shown that a strong TY-silver complex is formed, which at a ratio of 1:0.8 has no excess silver, does not decay in electrophoresis, and does not travel to the cathode (-).

**Figure 1:**
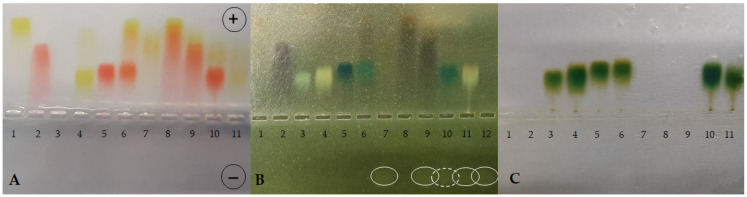
Agarose gel electrophoresis at pH 8.6 of TY, CR, Ag^+^, BSA and their combinations in different complexes (BSA-TY molar ratio was 1:10 and TY-Ag molar ratio was 1:1.6). (**A**): gel after electrophoresis in a veronal buffer; (**B**): reduction with sodium dithionite locating the presence of excess silver; (**C**): bromophenol blue-stained albumin; BSA free and in the complexes with CR, TY, or TY-Ag and CR-TY-Ag seen as migrating towards the anode; free Ag^+^ seen as migrating towards the cathode: (**1**) TY; (**2**) CR; (**3**) BSA; (**4**) BSA-TY (1:10); (**5**) BSA-CR (1:10); (**6**) BSA-CR-TY (BSA-CR = 1:10 and BSA-TY = 1:10; CR-TY = 1:2) visible additional streak of the complex CR-TY at the front; (**7**) TY-Ag (1:1.6); (**8**) CR-TY (1:1); (**9**) CR-TY-Ag (TY-Ag = 1:1.6); (**10**) BSA-CR-TY-Ag (BSA-CR = 1:10, BSA-TY = 1:10, TY-Ag = 1:1.6); (**11**) BSA-TY-Ag (BSA-TY = 1:10, TY-Ag = 1:1.6); (**12**) Ag^+^; (circles mark free silver moving towards the cathode (the dashed line indicates the smallest amount of free silver).

First, the complexes formed on the basis of the TY-Ag complex were analyzed in a molar ratio of 1:1.6 (Figure 1). BSA complexed with TY (sample 4) travels faster than free albumin (sample 3) (Figure 1C), and there is practically complete complexation of TY by BSA (Figure 1A). The addition of CR to BSA (sample 5) or to BSA-TY (sample 6) causes an additional increase in the rate of albumin migration in the complexes thus formed (Figure 1C). BSA complexes CR-TY (added in a molar ratio of 1:2), which is visible in sample 6. There is also an additional streak of the CR-TY complex migrating towards the anode (in the experiment where CR-TY was added in a ratio of 1:5, an excess of free TY migrating to the anode) (Figure 1A). BSA complexes CR-TY-Ag very well (Figure 1A, sample 10).

The TY-Ag complex (Figure 1A, sample 7) is formed, which travels more slowly than the free TY (sample 1), due to the positive charge of the complexed silver.

When comparing samples 8 (CR-TY), 9 (CR-TY-Ag), and 10 (BSA-CR-TY-Ag), migration to the anode is clearly slowed down as the complexes are increased by adding further factors. This proves the formation of these complexes (Figure 1A). Sample 10 shows the most effective silver binding (the dashed line in Figure 1B indicates the lowest amount of free silver in the sample).

**Figure 2:**
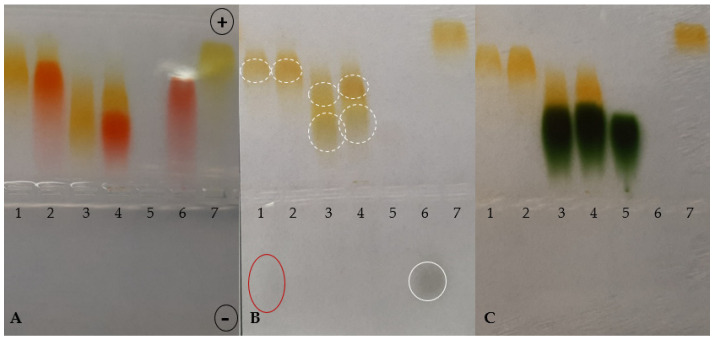
Agarose gel electrophoresis at pH 8.6 of TY, CR, Ag^+^, BSA and their combinations in different complexes (BSA-TY molar ratio was 1:10 and TY-Ag molar ratio was 1:0.8). (**A**): gel after electrophoresis in barbital buffer; (**B**): reduction with sodium dithionite locating the presence of excess silver; (**C**): bromophenol blue-stained albumin; BSA free and in the complexes with CR, TY or TY-Ag and CR-TY-Ag seen as migrating towards the anode; free Ag^+^ seen as migrating towards the cathode: (**1**) TY-Ag (1:0.8); (**2**) CR-TY-Ag (TY-Ag = 1: 0.8); (**3**) BSA-TY-Ag (BSA-TY = 1:10, TY-Ag = 1:0.8); (**4**) BSA-CR-TY-Ag (BSA-CR = 1:10, BSA-TY = 1:10, TY-Ag = 1:0.8); (**5**) BSA; (**6**) CR-Ag; (**7**) TY; a continuous line marks free silver moving towards the cathode, while the dashed line marks complexed silver.

Then, the complexes formed on the basis of the TY-Ag complex were analyzed in a molar ratio of 1:0.8 (Figure 2). Sample 1 shows a slight trace of unbound silver traveling towards the cathode, while the addition of CR in sample 2 completely binds the silver in the complex (CR-TY-Ag). CR increases the solubility of the TY-Ag complex (Figure 2B). The complexed silver is visible in both sample 3 (BSA-TY-Ag) and sample 4 (BSA-CR-TY-Ag). Due to the positive charge of Ag^+^, it binds to TY or TY-CR in albumin (Figure 2C). Non-protein bound TY-Ag complexes (samples 3 and 4) can also be observed, migrating faster than BSA-TY-Ag and BSA-CR-TY-Ag to the anode (Figure 2C). Albumin in these complexes travels to the anode faster than free BSA (sample 5) (Figure 2C). Congo red does not bind Ag^+^—migration towards the cathode is visible (sample 6) (Figure 2A).

As in the electrophoresis presented above, at the ratio TY-Ag = 1:0.8, slight traces of streaking excess silver were visible; therefore, the target ratio of TY-Ag = 1:0.5 was used in microbiological experiments. It ensures that the added silver is completely bound, and a strong complex is obtained that does not decompose under electrophoresis. Thereby it is certain that the administration of excess free silver in microbiological tests has been avoided.

#### 2.1.2. Albumin Complexes with TY and TY-Ag (UV/VIS Spectrophotometry)

**Figure 3:**
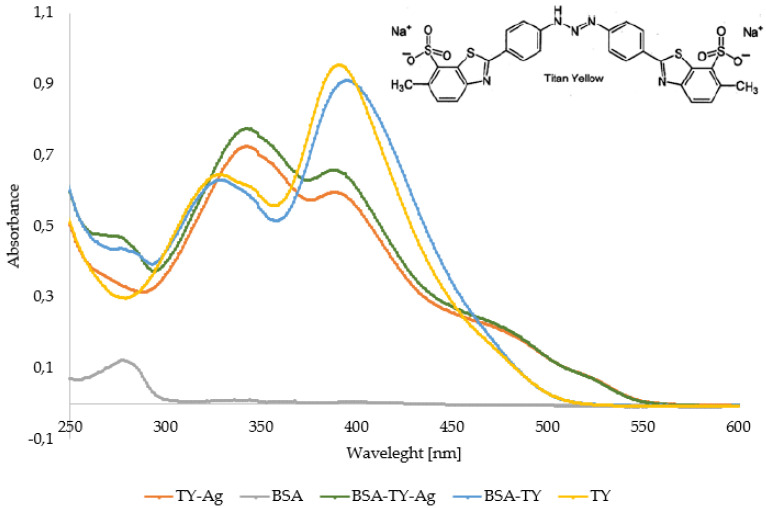
Comparison of UV/VIS spectra of TY, TY-Ag, BSA, BSA-TY, and BSA-TY-Ag. Changes in the spectrum in the emerging complexes are visible.

The comparative analyzes of the UV/VIS spectra show that TY binds to BSA, as evidenced by the hypochromic effect at 392 nm wavelength, with a slight shift towards longer wavelengths and maintaining the unchanged maximum at 330 nm. Additionally, there is a maximum at 280 nm related to albumin complexation. The spectrum of TY-Ag or BSA-TY-Ag differs markedly from the spectrum of free TY or BSA-TY complex (Figure 3).

#### 2.1.3. Albumin Complexes with TY and TY-Ag (Gel-filtration Chromatography on BioGel P-150)

**Figure 4:**
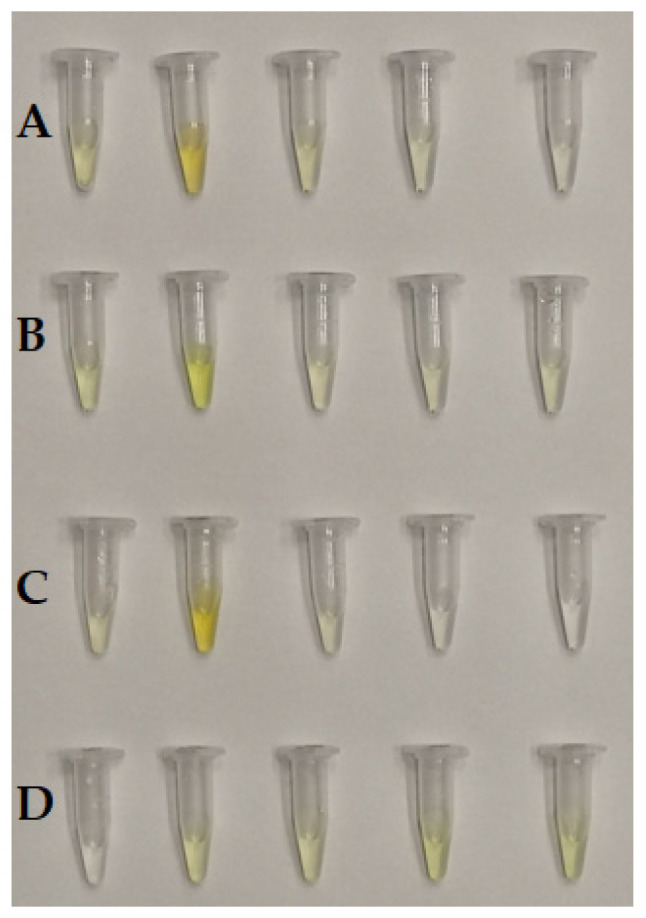
Successive samples (0.5 mL) collected after passing through BioGel P-150: (**A**): TY-Ag, (**B**): BSA-TY, (**C**): BSA-TY-Ag, (**D**): TY.

The analysis shows that both TY and TY-Ag are bound to albumin and descend quickly (approx. 1 mL each) the column filled with BioGel P-150. The free TY descends more slowly, becoming smoother as it passes through the gel, and most of it is delayed by about 2.5–3 mL. This demonstrates the complexation of TY and the TY-Ag system by albumin (Figure 4).

#### 2.1.4. Increase in Size of the TY-Ag Complex as Compared to free TY and Increase in Size of the TY-Ag Bound to Albumin (DLS Assays)

The autocorrelation curves of TY-Ag relative to free TY were analyzed. A shift in the autocorrelation curve of the TY-Ag complex relative to the autocorrelation curve of free TY was observed, which proves the presence of large TY aggregates formed under the influence of bound silver.

**Figure 5:**
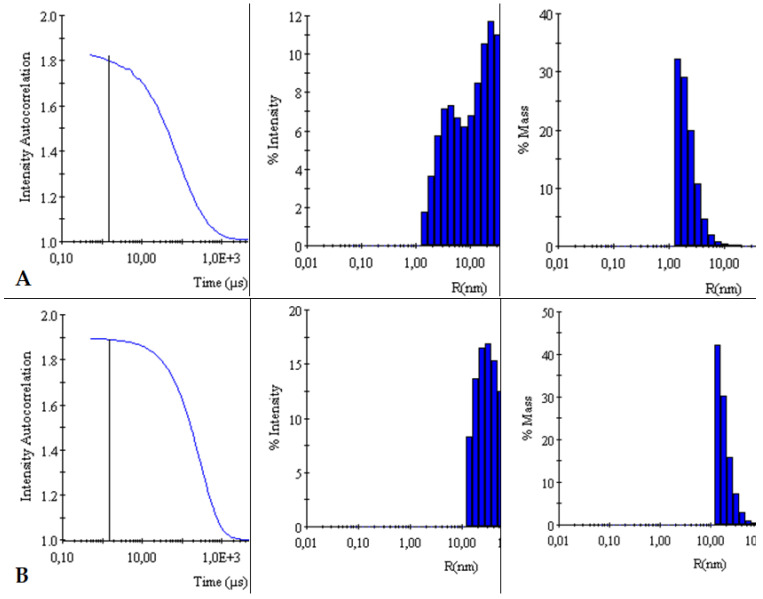
Comparison of intensity autocorrelation curves, % of intensity and % of the mass of: (**A**): TY and (**B**): TY-Ag.

This is also confirmed by the % intensity analysis. Visible polydisperse plot for free Titan yellow with two peaks (R_H1_ = 4.4 nm and R_H2_ = 26 nm) and monodisperse plot for the TY-Ag complex with one peak (R_H_ = 41 nm). During the % mass analysis, there is a clear shift of the maximum for the TY-Ag complex (R_H_ = 19.5 nm) compared to the free TY (R_H_ = 2.4 nm). This proves the presence of TY-Ag aggregates (Figure 5).

The BSA-TY autocorrelation curves against free albumin were analyzed. No change in the BSA versus BSA-TY autocorrelation curves was observed. This proves that TY is bound to a small degree by BSA, or that the whole thing is hidden in the pocket of albumin. On the other hand, a shift in the autocorrelation curve of the BSA-TY-Ag complex with respect to the autocorrelation curve of free BSA and BSA-TY was observed. This proves the presence of large BSA-TY-Ag aggregates.

**Figure 6:**
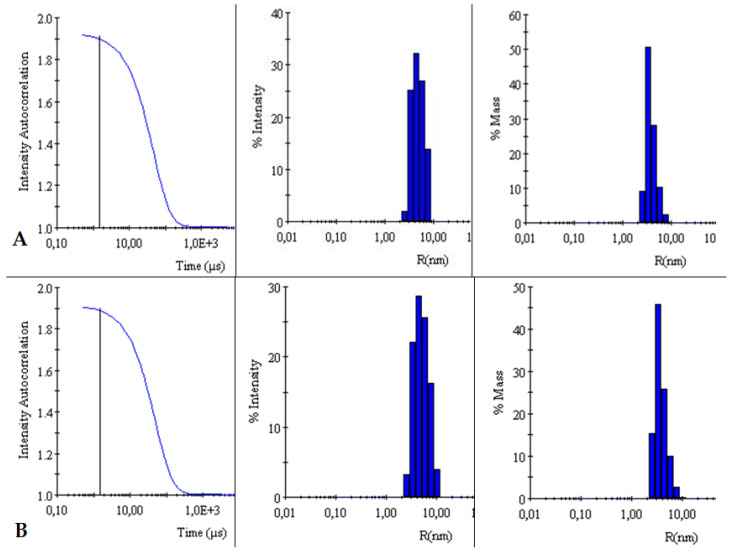
Comparison of intensity autocorrelation curves, % of intensity, and % of the mass of: (**A**): BSA and (**B**): BSA-TY.

Intensities and masses were also analyzed. Identical results were obtained for the samples of free BSA and BSA-TY complexes (in both cases for % intensity R_H_ = 5 nm, for % mass R_H_ = 3.9 nm). It was concluded that no large aggregates were formed and that TY was likely to bind at the albumin-binding cleft, and there was no bridging of albumin molecules (Figure 6).

**Figure 7:**
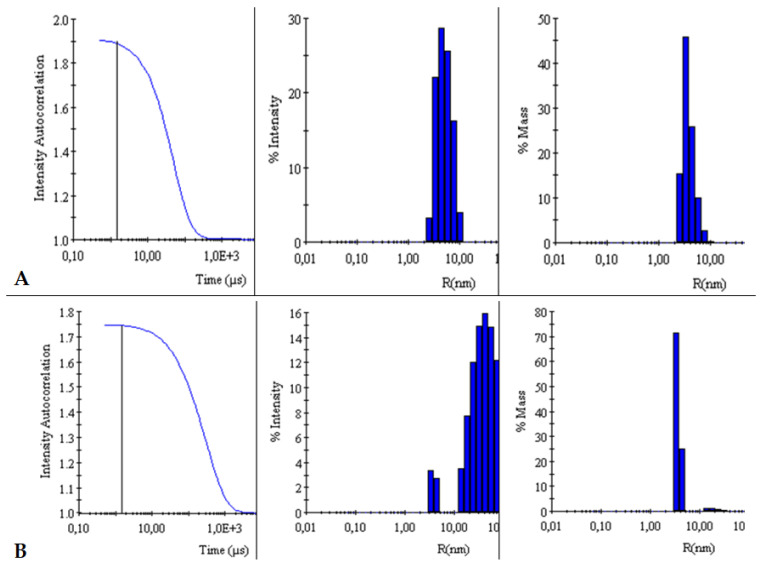
Comparison of intensity autocorrelation curves, % of intensity and, % of the mass of: (**A**): BSA-TY and (**B**): BSA-TY-Ag.

The autocorrelation curves of BSA-TY-Ag versus BSA-TY were also analyzed (Figure 7). A shift in the autocorrelation curve of the BSA-TY-Ag complex relative to the BSA-TY autocorrelation curve was observed, which proves the presence of large aggregates formed due to the binding of the Ty-Ag complex with albumin.

The appearance of an additional maximum was registered on the % intensity plot for the samples of the BSA-TY-Ag complex (R_H1_ = 3.8 nm and R_H2_ = 49.8 nm) relative to the maximum for free BSA and BSA-TY (in both cases for % intensity, there is only one R_H_ maximum = 5 nm). An additional maximum also appears in the % mass plot for the samples of the BSA-TY-Ag complex: R_H1_ = 3.6 nm and R_H2_ = 22.2 nm relative to the maximum for free BSA and BSA-TY (in both cases for % mass R_H_ = 3.9 nm). This proves the presence of larger BSA-TY-Ag aggregates or the possibility of Ag^+^-mediated bridging of two albumin molecules (BSA-TY-Ag-TY-BSA). As a result, nanosystems containing albumin and silver are created, which are still relatively small.

### 2.2. Microbiological Analysis

Analyzes were carried out to check whether the proposed silver-based system bound to the carrier in the form of TY or additionally bound to BSA can be used to effectively remove the bacterial film, with a simultaneous therapeutic effect on the tumor. The aim of the research was to determine the MIC and MBC of the analyzed systems. 

#### 2.2.1. No Inhibition of Bacterial Growth by free TY and Enhanced Inhibition of Bacterial Growth upon Complexation of TY with Ag^+^. *Escherichia coli* (EC, ATCC^®^ 35218™) and *Staphylococcus aureus* (SA, ATCC^®^ 29213™)

As a result of serial dilution of the samples, 4 samples were obtained in which the molar ratio of TY and silver was equal to 1:1 and the concentrations were as it shown in Table 1.

**Figure 8:**
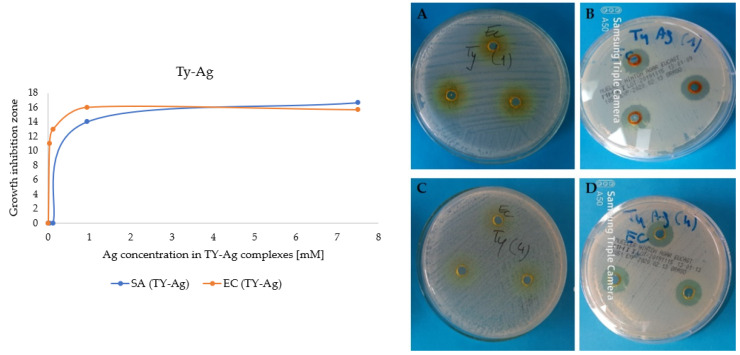
Graph: Growth inhibition zones of *E. coli* and *S. aureus* after the addition of different concentrations of Ag^+^ in the TY-Ag complex; (**A**): photos of plates: no growth inhibition zones after adding 7.5 mM of TY, or (**C**): 0.03 mM of TY; (**B**): growth inhibition zones after adding 7.5 mM of Ag^+^ complexed with 7.5 mM TY or (**D**): 0.03 mM Ag^+^ complexed with 0.03 mM TY.

Also 4 samples with analogous concentrations of free TY without silver were prepared. The zones of inhibition appearing after the addition of the TY-Ag complex were observed, while TY did not inhibit the growth of the analyzed bacterial strains (Figure 8).

#### 2.2.2. Determination of the Minimum Inhibitory Concentration (MIC) Value

**Figure 9:**
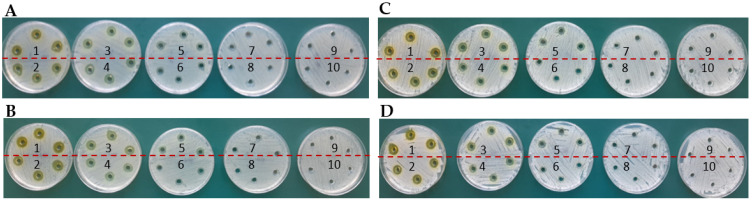
Determination of MIC by agar diffusion method for *E. coli* in the presence of complexes: (**A**): TY-Ag (1:0.5) (concentration No. 7: 0.019 mM; (**B**): BSA-TY-Ag (concentration No. 7: 0.019 mM; MIC determination for *S. aureus* in the presence of complexes: (**C**): TY-Ag (1:0.5) (concentration No. 7: 0.019 mM; (**D**): BSA-TY-Ag (TY-Ag 1:0.5; BSA-TY 1:10) (concentration No. 6: 0.039 mM). The ranges of silver concentrations (serial two-fold dilutions) were analyzed from 1.25 mM (No. 1) to 0.005 mM (No. 9). No. 10 is a control sample.

The value of MIC = 0.019 mM (for *E. coli* and for *S. aureus*) was determined for the molar ratio of TY-Ag (1:0.5), where we can be sure that the silver is completely bound (well diffusion in agar experiment). The presence of albumin in the complex doubles the MIC value 0.039 mM for Gram-positive *S. aureus*, which has a thick bacterial envelope, and there is no increase in the MIC value for Gram-negative *E. coli* (Table 2, Figure 9). 

#### 2.2.3. Determination of the Minimal Bacterial Concentration (MBC) Value

**Figure 10:**
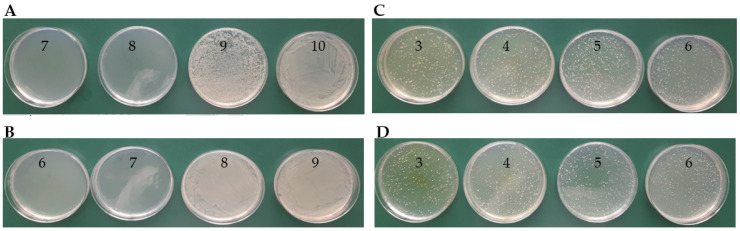
Determination of MBC on MHA agar plates for *E. coli* in the presence of complexes: (**A**): TY-Ag (1:0.5) (last of concentration No. 8: 0.036 mM); (**B**): BSA-TY-Ag (last of concentration No. 7: 0.07 mM); MBC determination for *S. aureus* in the presence of complexes: (**C**): TY-Ag (1:0.5) (above concentration No. 3: 1.12 mM—MBC not determined); (**D**): BSA-TY-Ag (TY-Ag = 1:0.5; BSA-TY = 1:10) (above concentration no. 3: 1.12 mM—MBC has not been determined). The ranges of silver concentrations (serial two-fold dilutions) were analyzed from 1.12 mM (No. 3) to 0.008 mM (No. 10).

MBC (Table 3) for *E. coli* was determined as 0.036 mM and 0.07 mM, respectively, for TY-Ag and BSA-TY-Ag (Figure 10A,B). For *S. aureus* the MBC in the concentration range assessed was not determined (Figure 10C,D).

#### 2.2.4. Increasing the Solubility of TY-Ag after Applying CR and/or BSA and Providing Protection, Improving the Antimicrobial Activity of Silver, as Shown by the Example of *E. coli* and *S. aureus*

The complexes with the TY-Ag molar ratio of 1:0.5 were analyzed, which, according to the results of the electrophoresis, were characterized by the complete binding of AgNO_3_ by TY. The concentration of silver in all analyzed samples was 4.7 mM, which was a value well above the MIC for both analyzed strains. Modified Kirby–Bauer method was used (agarose wells instead of disks).

**Figure 11:**
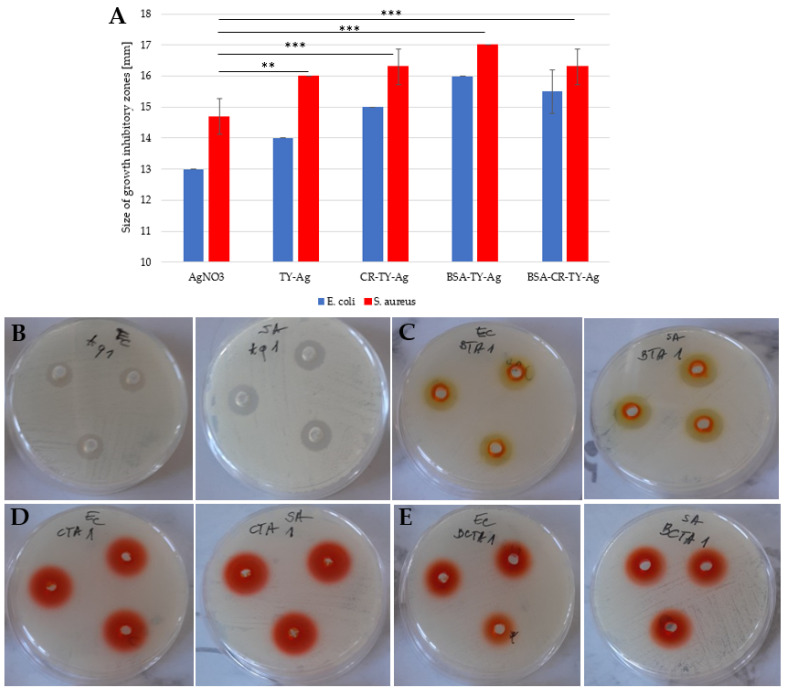
(**A**): A histogram presenting a comparison of the size of growth inhibitory zones of two strains: *Escherichia coli* (*E. coli*)and *Streptococcus aureus* after adding the silver complexed with: TY, CR-TY, BSA-TY and BSA-CR-TY; growth inhibitory zones on plates from left to right for *E. coli* and for *S. aureus* (each probe in triplicate) after adding: (**B**): AgNO_3_; (**C**): BSA-TY-Ag; (**D**): CR-TY-Ag, and (**E**): BSA-CR-TY-Ag (each probe in triplicate); concentration of silver in each probe: 4.7 mM. The graph shows the mean of the 3 experiments +/− standard deviation. The results were statistically analyzed using the Student’s *T*-test. Statistically significant differences (** *p* < 0.01; *** *p* < 0.001) in the size of growth inhibitory zones were observed between the AgNO_3_ samples and those to which the silver complexes were added.

Both in the case of *E. coli* and *S. aureus*, the influence of the presence of TY on the enlargement of growth inhibitory zones can be seen. Free AgNO_3_ produces smaller zones of inhibition than the complexed Ag^+^. An increase in these zones was also observed after the addition of CR and after the addition of BSA. However, the simultaneous use of BSA and CR (quadruple complex: BSA-CR-TY-Ag) resulted in smaller zones than using CR and BSA alone, but still increased relative to the free Ag^+^. The use of CR and/or BSA has been shown to increase the solubility of TY-Ag and to provide protection, thus improving the antibacterial effect of silver against both Gram-positive bacteria as exemplified by *S. aureus* and Gram-negative bacteria as exemplified by *E. coli* (Figure 11).

### 2.3. Cell Analysis

The effect of TY-Ag or BSA-TY-Ag complexes on T24 cell proliferation (MTS), migration (scratch test) as well as viability/survival, and apoptosis induction (Caspase 3 assay–Cas 3, Flow cytometry) was assessed. The preliminary results obtained by us earlier on human U937 lymphoid cell line in which late apoptosis (73.65% of cells) and necrosis (about 25% of cells) was observed after 72 h, after using TY-Ag 1:0.8 (where the concentration of TY was 0.072 mM and concentration of Ag^+^ was 0.057 mM [15] provided the basis for the analysis of the effect of these agents on bladder cancer cells.

#### 2.3.1. Reduction of T24 Cell Proliferation and Viability under the Influence of Ag^+^ Complexed with TY (TY-Ag) Similarly to BSA-TY-Ag (Visible Protective Role of Albumin)

**Figure 12:**
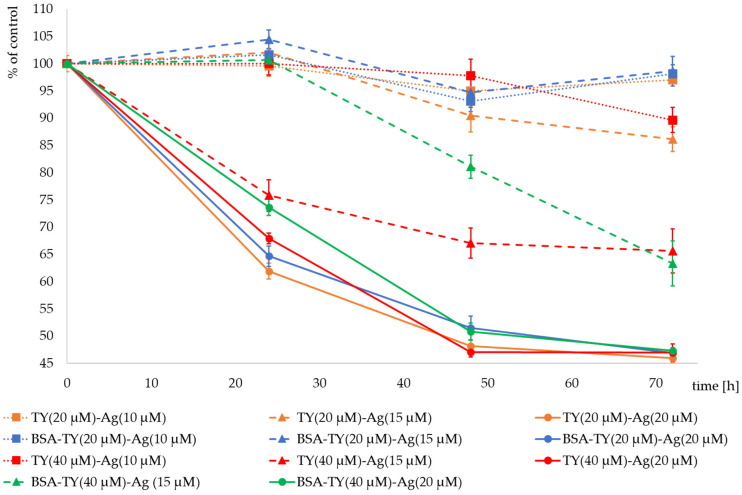
T24 cell proliferation expressed as % of control after addition of Ag^+^ (20 µM) (solid lines) complexed with TY (20 µM) (yellow), BSA-TY(20 µM) (blue), TY (40 µM) (red) and BSA-TY (40 µM) (green) separately; Ag^+^ (15 µM) (dashed lines) complexed with TY (20 µM) (yellow), BSA-TY (20 µM) (blue), TY (40 µM) (red) and BSA-TY (40 µM) (green) separately; Ag^+^ (10 µM) (dotted lines) complexed with TY (20 µM) (yellow), BSA-TY (20 µM) (blue) and TY (40 µM) (red) separately.

The MIC values were determined in the previously described experiment (for *E. coli* and *S. aureus* strains after the addition of TY-Ag in a molar ratio of 1:0.5) as the concentration of Ag^+^ = 0.019 mM (19 µM). The addition of BSA in both cases increased the MIC value to an average of 0.036 mM for both analyzed strains (clearly for *S. aureus* and less unambiguously for *E. coli*). Free TY gave no zones of growth inhibition. Hence, for the analysis of T24 bladder tumor cell proliferation, concentrations of TY = 40 µM and 20 µM were selected, with which complexes with Ag^+^ (TY-Ag = 1:0.5 molar ratio) and in some experiments additionally with albumin (BSA-TY = 1:10 molar ratio) were prepared. The complexes were added to the culture medium at the Ag^+^ concentrations of 10 μM, 15 μM, and 20 μM. The cell proliferation analysis was performed by the MTS assay at 24, 48, and 72 h after the addition of the agents. The exposure of T24 cells to TY-Ag resulted in significant dose-dependent suppression of proliferation, evaluated with MTS assay, compared to the control cultures (i.e., non-treated cells), as shown in Figure 12.

It was observed that an Ag^+^ concentration of 10 µM did not reduce cell proliferation in the presence of complexed TY (20 µM). BSA at these concentrations of TY and Ag^+^ had practically no effect on proliferation. The combination of Ag^+^ (10 µM) with TY at a higher concentration (40 µM) reduced the proliferation to 89.6% of the control after 72 h of culture.

Much greater differences are visible after using Ag^+^ at a concentration of 15 µM. The complex with a lower concentration of TY in TY(20 µM)-Ag(15 µM) reduces the proliferation of cells after 72 h of culture to the level of 86%, and the protective effect of BSA is visible, which maintains proliferation at the level of 98.6%. However, the higher concentration of TY in TY(40 µM)-Ag(15 µM) significantly reduces the proliferation of cells after 72 h of culture to the level of 65.6% of control. When cultures were observed after the cultivation time of 24 h and 48 h, the clear protective role of BSA was visible, which manifested by the increase of cell proliferation from 75.8% to 100% and from 67% to 81% of control after 24 and 48 h of cultivation, respectively. Only after 72 h of incubation of T24 cells in the presence of the TY(40 µM)-Ag(15 µM) complex, both with and without BSA, the same inhibitory effect on the proliferation rate was observed, equal to 65% of the control. It can be concluded that the concentration of Ag^+^ = 15 µM in complexes with TY is a limiting concentration, and by regulating the concentration of TY or BSA, one can obtain complexes reducing the proliferation to a greater or lesser degree, depending on the needs.

Silver at a concentration of 20 µM reduces the proliferation of cells both at the concentration of TY in the complexes equal to 20 µM and 40 µM. After 24 h of culture with TY(20 µM)-Ag(20 µM), proliferation drops to 63%. It is slightly elevated after using BSA in this complex. In contrast, in culture with TY(40 µM)-Ag(20 µM), proliferation decreased to 68%. BSA in this complex increases proliferation to 74%. After 72 h of cultivation with all the analyzed Ag^+^ (20 µM) complexes, the proliferation decreased to the level of approx. 45% of control. At this concentration of Ag^+^, a protective role of BSA was also observed for both the concentrations of TY used (20 µM and 40 µM) (Figure 12).

#### 2.3.2. Induction of Caspase-3 Activation in T24 Cells by TY-Ag

**Figure 13:**
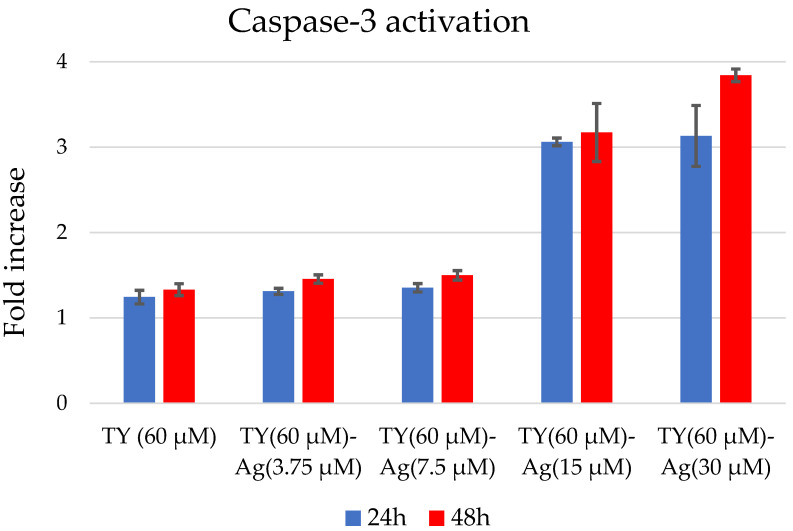
Caspase-3 activation after 24 and 48 h in T24 cells after TY-Ag stimulation.

The concentration of TY used in each sample was 60 µM (to check the TY effect at a dose minimal higher than the designated IC50), while the concentration of silver was variable. Concentrations of TY-complexed silver of 15 µM and 30 µM (TY(60 µM)-Ag(30 µM) = 1:0.5 molar ratio; TY(60 µM)-Ag(15 µM) = 1:0.25 molar ratio) caused Cas 3 activation, indicating apoptosis induction. The other concentrations of silver complexed with TY, namely 7.5 µM and 3.75 µM (TY(60 µM)-Ag(7.5 µM) = 1:0.125 molar ratio; TY(60 µM)-Ag(3.75 µM) = 1:0.625 molar ratio) had no effect of Cas 3 activation, just as free TY (Figure 13).

#### 2.3.3. Induction of Apoptosis in T24 Cells by TY-Ag (Flow Cytometry)

**Figure 14:**
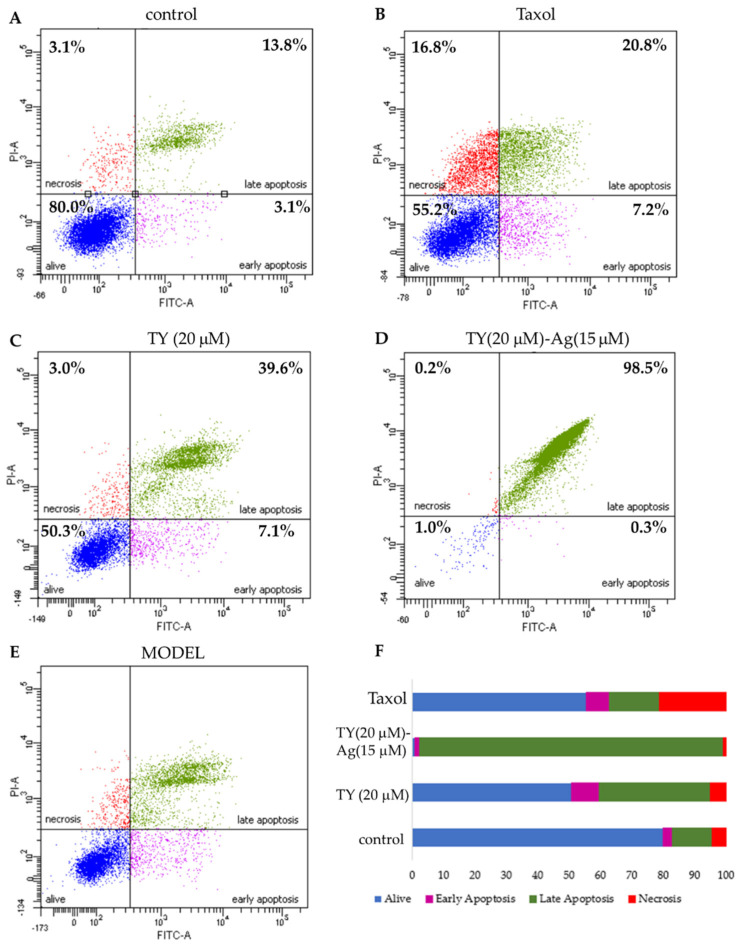
The effect of TY and TY-Ag complex on apoptosis and necrosis of T24 cells. The images show flow cytometry analysis of Annexin-V and PI staining presented in a dot-plot graph; (**A**): negative control; (**B**): positive control with taxol (50 nM); (**C**): T24 cells were incubated with TY (20 µM concentrations), (**D**): TY-Ag (TY 20 µM and Ag^+^ 15 µM concentrations); (**E**): graphic representation of four cell states: alive - the lower-left square; cells undergoing necrosis—the upper left square; cells in early apoptosis - the right lower square; and cells in late apoptosis the upper right square; (**F**): Cumulative bar charts show the inter-relation between the state of T24 cells after 48 h exposure to TY (20 µM), TY(20 µM)-Ag(15 µM), and taxol.

Using the flow cytometry method (Annexin/propidium iodide), apoptosis was assessed in the tumor cells of the bladder cancer cell line T24 after the addition of the TY(20 µM)-Ag(15 µM) complex. A total of 48 h after the addition of the factor, TY-complexed silver, added at a concentration of 15µM, resulted in a nearly complete apoptosis of 96.6%. This result confirmed that this concentration, close to the MIC value = 0.019 mM (for both analyzed *E. coli* and *S. aureus* strains) for silver in the TY-Ag complex (1:0.5), can induce complete apoptosis of T24 cells (Figure 14).

#### 2.3.4. Reduction of T24 Cell Migration by TY-Ag (Scratch Assay)

**Figure 15:**
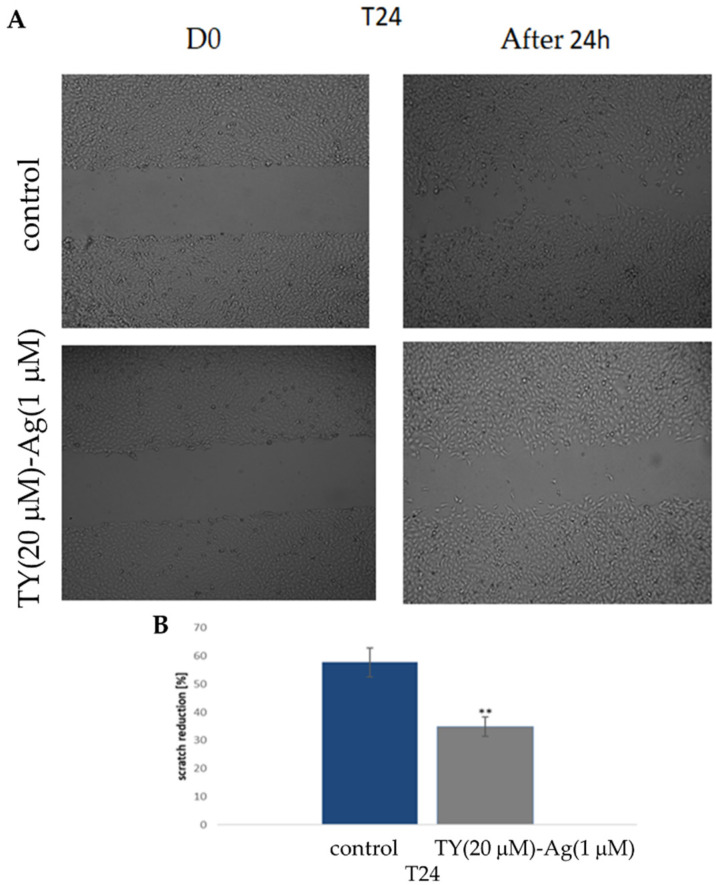
The effect of TY(20 µM)-Ag(1 µM) on the motility of T24 cells (**A**): exposure to TY(20 µM)-Ag(1 µM) inhibited migration of T24 cells in a scratch assay observed after 24 h; (**B**): percentage of scratch reduction after 24 h (*n* = 3).

The T24 cell lines were treated with TY(20 µM)-Ag(1 µM) complex, and the cell migration was evaluated with a wound-healing test/scratch assay. A significant reduction in cell motility was observed upon exposure to TY(20 µM)-Ag(1 µM) (Figure 15). Optical images show scratch overgrowth with time for T24 cells (Figure 15A). The photographs were captured immediately after wounding as well as 24 h later with the corresponding control (non-treated cells). Non-treated cells almost entirely closed the wound in approximately 24 h while the scratch remained open in cells treated with TY(20 µM)-Ag(1 µM). These results indicate that the motility of the T24 cell line was significantly inhibited by the presence of TY(20 µM)-Ag(1 µM) after 24 h. The percentage effect of scratch reduction is shown in Figure 15B.

#### 2.3.5. Increasing the Number of Cells in the G1 Phase and Reducing the Number of Cells in the S and G2 Phase in T24 Cells under the Influence of the Tested Compounds: TY, TY-Ag, BSA-TY and BSA-TY-Ag (Flow Cytometry/Cell Cycle Analysis) 

**Figure 16:**
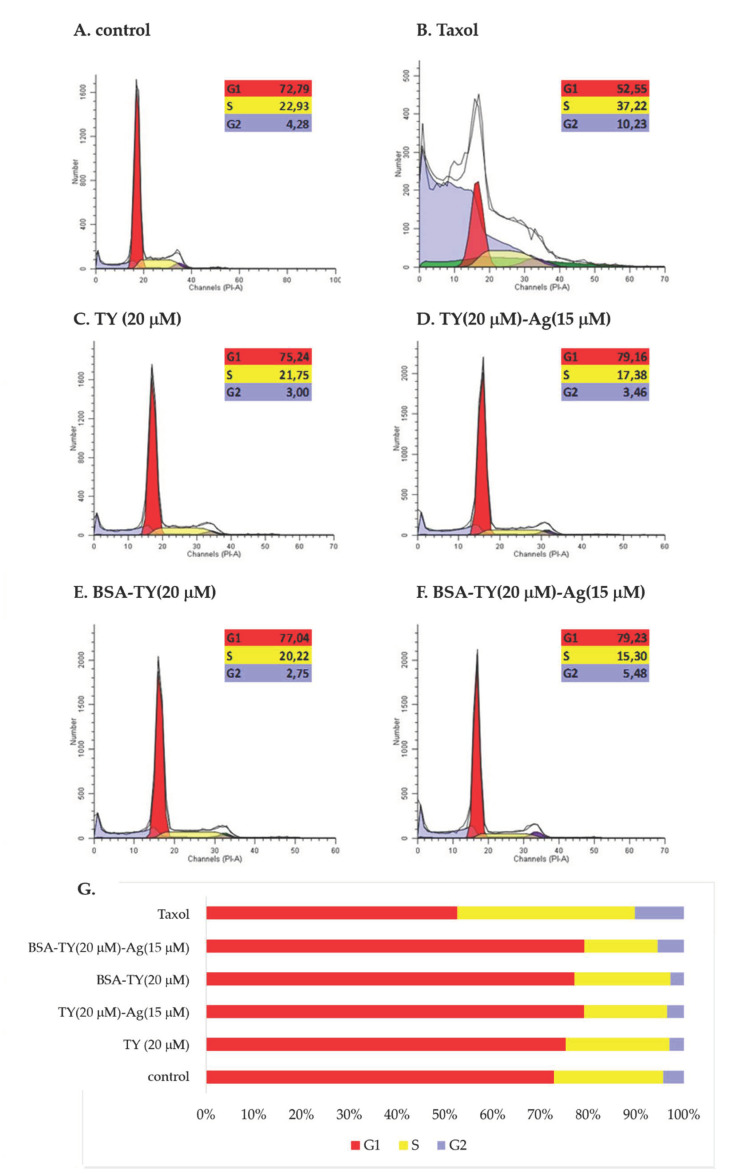
The effect of (**B**): taxol, (**C**): TY, (**D**): TY-Ag, (**E**): BSA-TY, and (**F**): BSA-TY-Ag on the cell cycle of T24 cells. (**A**): control; (**G**): percent of cells at G1, S or G2 phase of cell cycle.

The arrest of cell division may be the result of disturbances in the course of the cell cycle and/or induction of apoptosis and necrosis in cells. Therefore, in the next stage of the study, the influence of the tested compounds on the course of the cell cycle in the cultures of T24 cells after 48 h of incubation was determined. Cytometric analysis showed that the tested compounds (except for taxol) caused a slight increase in the number of cells in the G1 phase while reducing the number of cells in the S and G2 phases of the cell cycle (Figure 16).

## 3. Discussion

The prevention of infections is mainly associated with the use of antibiotics, the effectiveness of which is decreasing. In addition, frequent and recurring infections can cause cancer. For these reasons, finding an alternative, innovative therapy integrating antibacterial and anti-cancer treatment is the goal of scientific research [5,27,28].

Finding an agent that would combine the ability to minimize infections and anti-cancer activity seems to be very important. Such an agent could have practical applications in hospital treatments, and such attempts have long been made, but most involve alternative use of antibiotics. Various antibiotics are used in the treatment of neoplasms due to their anti-proliferative, pro-apoptotic, and anti-epithelial-mesenchymal transition (EMT) abilities [29]. Yadav et al. described fluoroquinolones which have been used as antibiotics for over four decades. Recent research highlighted their use as pharmacological compounds with multifaceted implications. Repositioning of fluoroquinolones into anti-cancer molecules seems to be a highly plausible option owing to their profound immunomodulatory, pro-apoptotic, anti-proliferative, and anti-metastatic potential [30]. Research conducted by Kamat et al. described the use of antibiotics ciprofloxacin, trimethoprim-sulfamethoxazole, cefazolin, or nitrofurantoin in bladder tumor treatment [31]. However, the newest research showed that antibiotics could also induce cancer by disrupting the gut microbiota, which further promoted chronic inflammation, altered normal tissue metabolism, led to genotoxicity, and weakened the immune response, thereby adversely affecting cancer treatment [32,33].

Among non-antibiotic anti-infectious agents, silver has been known for a long time for its antibacterial and anti-inflammatory properties. Applied in the form of nanosilver, it is used in many areas of medicine. Plastic catheters coated with silver nanoparticles prevent biofilm formation by bacteria, including *E. coli* and *S. aureus*. It is used to produce disinfectants, in dental composites, or as bactericidal coatings [34]. Silver is even used to prevent HIV binding to host cells [35]. The antimicrobial effects of silver are mostly attributed to silver ions [36,37]. Silver nanoparticles (AgNPs) continuously release silver ions in an aqueous microenvironment [38,39]. Due to the larger surface of AgNPs, they show stronger and better bactericidal activity [40]. The main reasons for the bactericidal properties of AgNPs are the disruption of the integrity of the bacterial cell by binding to the basic cellular structures [41,42], especially to their -SH groups [43]. AgNPs also generate reactive oxygen species (ROS) and free radicals that damage the bacterial cell wall and inhibit respiratory enzymes [44], as well as disrupt DNA replication and destroy bacteria. Silver nanoparticles are biocidal against various Gram-positive and Gram-negative bacteria [45].

AgNPs exhibit anti-cancer properties against various cancer cell lines, such as MCF-7 breast cancer cells [46], HCT116 colon cancer cells [47,48], prostate cancer cells [49], HeLa cells [50], or lung carcinoma A549 cells [51]. AgNPs can be used to induce regression of bladder cancer through apoptosis under the influence of ROS-producing radicals [52,53]. However, there are reports on a higher toxicity profile of silver nanoparticles than gold nanoparticles for normal organs. It relates to the high ROS generation capacity [54]. This property of AgNPs has so far represented a significant limitation in the development of these nanoparticles as drug delivery systems. However, recent studies have shown their biocompatible nature after coupling or coating with polymers. In our research, we also assumed that the conjugation of silver with a supramolecular compound, in particular Titan yellow, and additionally with CR or with albumin, will increase its biocompatibility.

Titan yellow comprises symmetric polar groups and aromatic rings. Its halves are linked by a tri-azo bond capable of complexing metal ions, particularly silver and mercury. The silver-containing complex is more convenient due to its stability and formation in both neutral and slightly alkaline environments [15,55,56]. Thanks to this binding, Ag^+^ becomes better available both for bacteria to fight them and for bladder cancer cells. Our research, based on electrophoretic methods, gel chromatography, and UV/VIS spectrophotometry, also showed that the TY-Ag system could additionally be bound by albumin or by Congo red. As a result, complexes were obtained that not only bound ionic silver but also provided protection and better solubility. The TY-Ag complexes and the triple complexes of BSA-TY-Ag or CR-TY-Ag represent systems of potential therapeutic value. Due to the very good solubility provided by CR, as well as the protective effect of albumin, these systems can reduce the accumulation of metals in the body by facilitating the removal of silver from the complex. There are examples in the literature of the interaction of silver nanoparticles with proteins. Although the capping proteins can increase the internalization of AgNPs into cells via endocytosis, regulate cellular uptake and bioavailability of AgNPs, they can also be responsible for the toxic activity of AgNPs [57]. The interaction of AgNPs with albumin has been described [58,59]. The silver complexes with TY or BSA-TY presented in this paper give an opportunity to create high-capacity systems. The pursuit of high payload combined with high targeting efficiency will increase the localization of cancer drugs, thus avoiding toxicity and side effects caused by drugs being distributed outside of the target.

Therefore, further studies are needed to understand the type of proteins and their precise role in the formation and bioactivity of nanoparticles.

Microbiological analyzes showing the effect of silver in various forms have been previously carried out on different bacterial strains [60]. Nevertheless, the MIC and MBC values after treatment with TY-Ag or BSA-TY-Ag in *E. coli* and *S. aureus* have been presented here for the first time. *E. coli* was selected because it is the main bacteria isolated from cases of urinary tract infections [23], whereas *S. aureus*, a facultative anaerobic Gram-positive coccus, was selected for use in this study because it is one of the facultative bacteria found in inflammation of the urinary bladder and it can develop resistance to antimicrobial agents. *S. aureus* may be a pathogen of the urinary tract among patients staying in long-term care facilities, wearing indwelling catheters, with urinary tract obstruction and/or malignant neoplasms [24].

Since our research has shown that free TY does not inhibit the growth of *E. coli* or *S. aureus* bacteria, while zones of growth inhibition are visible with the Ag^+^ complex, TY can be considered a useful type of carrier for silver. The results obtained by us have shown that thanks to the complexation of Ag^+^ with TY, a lower concentration of the administered silver can be used, which significantly reduces its toxicity and, additionally, provides protection. Additional protection is provided by albumin, which in the BSA-TY-Ag complex has increased the zones of inhibition of bacterial growth in both *E. coli* and *S. aureus*, as confirmed by our research. Additionally, the complexes with Congo red, which increases the solubility of the TY-Ag complex, increased the zones of inhibition of bacterial growth. The use of CR and/or BSA has been shown to increase the solubility of TY-Ag and to provide protection, thereby improving the antibacterial activity of silver in both Gram-positive bacteria, for example *S. aureus*, and Gram-negative bacteria, e.g., *E. coli*.

The relationship between the size of nanosystems and their antimicrobial efficacy (prokaryotic cells) is often analyzed in the literature. Similar to prokaryotic cells, in eukaryotic cells (antitumor efficacy), the size of AgNPs is a key factor determining their penetration into the cells by diffusion (translocation), endocytosis, or phagocytosis [61,62,63]. In recent times, it has been established that particle size is particularly important while other physicochemical parameters are under control. To confirm this, a systematic evaluation of the size-dependent biological profile and biodistribution of the three monodisperse drug-silica nanoconjugates at 20, 50, and 200 nm was performed in a previous study. This evaluation was performed through laboratory experiments in conjunction with mathematical modeling to determine the optimal size of the most effective anti-cancer drug delivery system. Thanks to this study, it was found that 50 nm drug-silica nanoconjugate particles exhibited the highest retention in cancer tissue over time, leading to deeper tissue penetration and effective internalization in cancer cells along with slower removal [64]. Silver in a nanometric scale (less than 100 nm) has different antibacterial properties compared with the bulk form of silver metal, like a large effective surface area of individual silver nanoparticles and strong toxicity to a wide range of microorganisms [65]. The analyzes carried out in this study with the use of DLS showed that the hydrodynamic diameter of the TY-Ag complex is about 40 nm, while the BSA-TY-Ag aggregate is about 40–50 nm. As a result, nanosystems containing albumin and silver are created, which are relatively small in size, which is very advantageous in the context of delivering silver to tissues.

Different silver-based nanosystems may give different MIC and MBC results for the tested bacterial strains. This variability may be due to the methodology used to prepare the silver nanoparticles and the size of the silver nanoparticles used. A study by Ayala-Núñez NV et al. estimated MIC, MBC of 10 nm silver nanoparticles at a concentration of 1.35 mg/mL against *S. aureus*. [66]. The authors indicated that the ultra-fine particle size caused it to function at a lower concentration. A commercially available 5 nm silver nanoparticle was used in that study. In a study by Agnihotri et al., silver nanoparticles smaller than 10 nm showed enhanced antimicrobial activity. They also concluded that compared to other sizes of silver nanoparticles, the 5 nm size has the fastest antibacterial activity [67].

Based on these results, we also conducted a MIC and MBC analysis of our test systems to determine the antimicrobial efficacy of our 50 nm nanosystems. MIC values for TY-Ag (1:0.5) were estimated at 0.019 mM (for *E. coli* and for *S. aureus*), and for BSA-TY-Ag at 0.019 mM for *E. coli* and 0.039 mM for *S. aureus*. MBC for TY-Ag (1:0.5) was found to be 0.036 mM (for *E. coli*) while the determination of MBC for *S. aureus* was impossible. MBC for BSA-TY-Ag was calculated as 0.07 mM(for *E. coli*), whereas the determination of MBC for *S. aureus* was impossible. It was reported that the activity of silver in AgNPs against Gram-negative bacteria was stronger than against Gram-positive bacteria [68,69,70,71], which was confirmed by the results of the present study. It is due to the presence of a thicker peptidoglycan cell wall in Gram-positive bacteria. However, there are works that contradict this statement [72,73,74] or show variable susceptibility within these bacterial groups [75,76]. The present results demonstrate the inhibitory effect of bound albumin on the activity of complexed silver in bacteria. The addition of BSA increases the MIC for *S. aureus*, also increases MBC for *E.coli* (for *S. aureus*, it was not possible to perform such an observation due to the inability to determine the MBC). BSA also provides protection in bladder cancer T24 cell culture (reduces the cytotoxic effect of the TY-Ag system). It can be treated as an advantage if we consider the system with albumin as a drug carrier that will prolong the action of silver, causing its gradual release. This protein may also play a protective role, e.g., when administered intravenously.

Comparing our results with the results of some research teams, we noticed low values of the above parameters (at the level of a few µM), much lower than those obtained with the use of biologically or chemically synthesized silver nanoparticles [77]. In our research, we obtained nanosystems with a size of about 40–50 nm, with an intense antibacterial effect obtained at concentrations of 3 µg/mL (19 μM). These results are comparable to the results of Wypij M. et al. They obtained nanoparticles AgNPs, which were spherical in shape, small in size (mean 13.2 nm), and exhibited antibacterial activity against both Gram-positive and Gram-negative bacteria (MIC of 8–128 μg/mL, MBC of 64–256 μg/mL). However, *S. aureus* was much less sensitive (MIC = 128 μg/mL and MBC = 256 μg/mL) than Gram-negative bacteria *E. coli* (MIC values of 8 μg/mL; MBC values 32 μg/mL), which is different from the results obtained by us [63]. Therefore, our findings are in line with these previously published data, which is extremely important due to the lower efficient doses of silver.

Despite the strong antimicrobial activity of AgNPs and a wide range of biomedical applications, their use as therapeutic agents is limited due to their cytotoxicity to mammalian cells [78].

According to [79], if the cell viability was reduced to <70% of the control, the agent would have a cytotoxicity potential. In our study, the viability of T24 cells after 24 h was higher than 70% when the cells were stimulated with TY(20 µM)-Ag, TY(40 µM)-Ag and BSA-TY(20 μM)-Ag at an Ag^+^ concentration up to 15 μM. At the same time, among the tested bacteria, MIC values for TY-Ag were lower for Gram-negative bacteria, such as *E. coli,* as well as for Gram-positive bacteria, such as *S. aureus* (MIC = 19 μM). The use of the BSA-TY-Ag complex resulted in no changes in MIC values for Gram-negative bacteria, such as *E. coli* (MIC = 3 μg/mL), and resulted in higher MIC value for Gram-positive bacteria, such as *S. aureus* (MIC = 6 μg/mL). This result in the case of Gram-positive bacteria is more favorable than that obtained in the studies of Wypij M. et al. They investigated the cytotoxic effect of the silver biogenic AgNPs nanoparticles biosynthesized by strain SF23 on MCF-7 breast cancer cells and RAW 264.7 macrophages. The cell viability of both tested cell lines was significantly inhibited in the presence of AgNPs at a concentration of 16 μg/mL or higher, which gives similar results as those obtained for silver in our complex presented in this study on the cells of bladder cancer T24, with a significant degree of malignancy. AgNP concentration in the range of 16–64 μg/mL decreased the viability of RAW 264.7 cells from 42.2 to 14.2%, while in the case of MCF-7 cells, the viability of the measured cells decreased from 38 to 15.8% [63]. Based on these findings, we are aware that there is a need to search for concentrations of the tested TY-Ag complexes, which will be microbicidal, decrease in T24 cells proliferation, and will not be cytotoxic to normal cells at the same time. Our results show that the tested Ty-Ag complex can be considered as a potential antimicrobial agent, which exhibits antibacterial activity against Gram-negative and Gram-positive bacteria at doses being safe for healthy cells.

It is worth noting that the conducted research also showed the inhibition of cell migration under the influence of TY-Ag, which may be of great importance when it comes to metastasis. In addition, studies have shown the cytostatic and cytotoxic effects of the system under study on neoplastic cells. It appears obvious that in order to effectively eradicate tumors, it is necessary to trigger apoptosis of cancer cells. Our preliminary results for TY-Ag on human U937 lymphoid cell line cells confirm the efficacy of Ag^+^ complexed at 9.8 μg/mL. Late apoptosis (73.65% of cells) and necrosis (about 25% of cells) were demonstrated after 72 h, after using TY-Ag 1:0.8 (where concentration of TY was 0.072 mM and concentration of Ag^+^ was 0.057 mM [15]. Zhou et al. described apoptosis of T24 cells induced by 48 h exposure to silver complexed in the Ag-SP-DNC complex (20 µM) at the level of 28.72% (early and late apoptosis together) [80]. Our results obtained for TY-Ag (1:0.8) showed a strong pro-apoptotic effect of this system (late apoptosis after 48 h affected 96.6% of cells) at concentrations of TY(20 µM)-Ag(15 µM), confirmed by a modified Cas3 test. It is worth noting that the activation of Caspase-3 by free TY at a concentration of 60 µM was at a negligible level close to the control sample one. 

To summarize, we have demonstrated for the first time that TY-Ag complex regulates proliferation, survival, and migration of T24 urinary bladder cells as well as inhibits bacterial growth of two reference strains, *E. coli* and *S. aureus,* showing the high antibacterial activity in relatively low concentration (19 μM). TY-Ag complexes were characterized and analyzed for their antibacterial and cytotoxic activities. They can interact with protein, in particular albumin, which increases the biocompatibility and protects the system, or with Congo red, a supramolecular carrier that can provide for targeted transport. The TY-Ag complex is a promising compound for the treatment of bladder cancer as well as the treatment of bactericidal infections and could open up new avenues for new non-antibiotic treatment strategies for infections.

## 4. Materials and Methods

### 4.1. Materials

Titan yellow (TY, 99% purity, WARCHEM, Warsaw, Poland), Congo Red (CR, 96% purity, Aldrich Chemical Company, Inc. Milwaukee, WI, USA), silver nitrate (Sigma Aldrich, Co., St. Louis, MO, USA), bovine serum albumin (BSA, 96% purity, Sigma-Aldrich, Co., St. Louis, MO, USA) were used.

Reference strains recommended by EUCAST (European Committee on Antimicrobial Susceptibility Testing) were chosen among Gram-positive strains: *S. aureus* (SA, ATCC^®^ 29213™) and among Gram-negative strains: *E. coli* (EC, ATCC^®^ 35218™). American Type Culture Collection (ATCC, Manassas, VA, USA) delivered these two prokaryotic bacteria strains.

Bladder cancer cell line (T24, HTB4) was purchased from ATCC (American Type Culture Collection, Manassas, VA, USA). MTS proliferation test (Promega, Madison, WI, USA), assay for activity of Caspase-3 (DEVD-like caspase activity, Gentaur Ltd., Sopot, Poland ), Annexin V with propidium iodide for Flow Cytometry analysis (BD Biosciences, San Jose, CA, USA) and PI/RNase Staining Buffer assay (Becton Dickinson, New York, NY, USA) were used to analyze the influence of the tested factors on the cells.

### 4.2. Methods

#### 4.2.1. Synthesis of Silver Complexes with TY (TY-Ag), with Congo Red (CR-TY-Ag), with Albumin (BSA-TY-Ag), or with Albumin and Congo Red (BSA-CR-TY-Ag)

TY and CR were dissolved in 0.05 M TRIS/HNO_3_/0.9% KNO_3_ buffer, pH 8.2 to the appropriate concentration. It was boiled for 2 min at 100 °C to dissolve, then cooled. Initially, the molar ratio CR:TY = 1:2 (and 1:5—unpublished results) was calculated.

AgNO_3_ was prepared fresh in a dark tube. It was dissolved in 0.05 M Tris/HNO_3_/0.9% KNO_3_ buffer, pH 8.2. Initially, the molar ratios of TY-Ag = 1:1.6 (samples with excess silver) or 1:0.8 (samples with all silver bound) or 1:0.5 (samples with completely bound silver used in microbiological experiments to completely eliminate free silver) were calculated. To prepare the TY-Ag complex, AgNO_3_ solution was added to TY and incubated for 3 min.

To prepare complexes with albumin, a 10× excess of TY over BSA or a 10× excess of CR over BSA was initially calculated. Then, after addition of BSA and the buffer, the mixture was incubated for 10 min. Co-micelle CR-TY (both components with the same molar mass) was used at 12-fold excess over BSA.

#### 4.2.2. Characterization of TY-Ag Complexes with Protein or with CR (BSA-TY-Ag, BSA-CR-TY-Ag)

- Electrophoresis

Electrophoresis was performed in 1% agarose gel (0.06 M sodium barbital buffer pH 8.6). Proteins were fixed in the gel with picrate and then stained with bromophenol blue solution. CR was removed from the gel by reduction with sodium dithionite. The sodium dithionite also revealed the location of the silver after the separation. The differences in the speed of migration of individual complexes and the possibilities of silver binding depending on the molar ratio of TY-Ag (1:1.6 vs. 1:0.8) were assessed.

- Assessment of TY and TY-Ag binding by BSA—Spectrophotometry

The synthesis of the TY-Ag complex was observed visually by the color change of the TY from yellow to orange after adding AgNO_3_ and spectroscopically using UV/VIS spectroscopy (CarryWinUV, Perlan, Agilent Technologies, Inc., Santa Clara, CA, USA) in a wavelength range from 200 to 800 nm, at a resolution of 1 nm. The TY without silver nitrate was used as the control sample. The spectra of TY, TY-Ag, as well as BSA-TY and BSA-TY-Ag, were compared, and on that basis, the formation of complexes was assessed.

- Assessment of TY and TY-Ag binding by BSA - Gel filtration Chromatography

Using the gel-filtration method, the speed at which the systems differing in molecular weights (TY and the BSA-TY complex in a molar ratio of 1:10) passed through the column filled with BioGel P-150 was compared. The separation was done in 0.01 M PBS pH 7.4 buffer. An 80µL sample was applied to the column filled with 4 mL of the BioGel P-150, then the separation was carried out. Spectrophotometric analysis of the migration rates of free TY, TY-Ag, and complexed with protein was performed. An observed low value of the elution volume indicates the formation of large and stable complexes.

- DLS

Measurement and comparison of the hydrodynamic radii of TY and TY-Ag, BSA and BSA-TY or BSA-TY and BSA-TY-Ag were carried out with a Dyna Pro dynamic light scattering (DLS) detector (Wyatt Technology, Santa Barbara, CA, USA) with an incident laser beam at λ = 824 nm and a fixed scattering angle of 90°. Measurement of each sample was performed at 25 °C after 3 min incubation in the DLS instrument. Each sample was measured 4 times with 20 acquisitions lasting 10 sec each. Outliers were rejected from the analysis, and the results were averaged.

#### 4.2.3. Antibacterial Assays

- Microorganisms

In order to evaluate the effect of silver complexed with TY or with the CR-TY or with BSA-TY systems, screening tests were performed on two model reference strains: Gram-positive and Gram-negative bacteria. The complexes were tested using strains from the collection of the American Type Culture Collection (ATCC). In the case of *S. aureus* and *E. coli*, reference strains recommended by EUCAST (European Committee on Antimicrobial Susceptibility Testing) were chosen as control strains for susceptibility testing and as control strains in quality control. These strains are *S. aureus* (ATCC^®^ 29213™) and *E. coli* (ATCC^®^ 35218™)—a strain producing TEM-1β-lactamase, which causes resistance to all β-lactam antibiotics.

- MIC determination. Well Diffusion in agar. Assay and growth conditions

The minimum dilutions of the analyzed compounds that inhibited the growth of the microorganisms were denoted as minimum inhibitory concentration (MIC). The values of MIC for different kinds of complexes, TY-Ag and BSA-TY-Ag; for two kinds of strains, *E. coli* (EC, ATCC^®^ 35218™) and *S. aureus* (SA, ATCC^®^ 29213™) were determined with the method of well diffusion of compounds in agar. For this purpose, appropriate bacterial suspensions were prepared according to the following methodology presented by Gibała et al.: 0.5 optical density (OD) in McFarland scale was made to receive 1.5 × 10^8^ CFU (colony forming units)/ mL solution, then the cell suspension was diluted to 10^6^ CFU mL^−1^. The prepared suspension was used for further studies, including the determination of MIC. The modified diffusion-disk screening method described by Gibała et al. was used to study the antimicrobial efficacy of TY-Ag (molar ratio 1:0.5) (or its complexes with BSA) by evaluating the visible growth of microorganisms in the agar [81]. Serial two-fold dilutions of the analyzed compounds in concentrations listed in Table 4 were used.

- MBC determination

The values of minimum bactericidal concentration (MBC) for different kinds of comlexes, TY-Ag and BSA-TY-Ag; for two kinds of strains, *E. coli* (EC, ATCC^®^ 35218™) and *S. aureus* (SA, ATCC^®^ 29213™) were determined according to the method described by Gibała et al. [81].

#### 4.2.4. Cytotoxicity Assays

- Cell Culture

T24 human bladder cancer cells were used in the study. These are transitional cell carcinoma (TCC) cells. Cells were grown in McCoy’s culture medium (BioWest, Riverside, MO, USA) containing penicillin (100 U/mL), streptomycin (100 μg/mL), and 10% fetal bovine serum (FBS, EURx Sp. z o.o., Gdańsk, Polska). Cultures were carried out at 37 °C with 5% CO_2_ content and 95% humidity, changing the medium every 24–48 h. Cells were grown until the culture area was completely covered. Cells were then passaged using 0.05% trypsin.

- Treatments of the Cells

The Titan yellow (TY) complexed with silver (TY-Ag) and TY-Ag in combination with albumin (BSA) were added at 24 h from seeding the cells on the plate. The combination of 3 concentrations of Ag^+^ compound: 10 μM, 15 μM, and 20 μM and 2 concentrations of Titan Yellow (TY): 20 μM and 40 μM were analyzed in the cell proliferation assays. Then the selected concentrations were used for the evaluation of cell survival and migration.

- Cell Proliferation Assays

T24 cells were seeded on 24-well plates at a density of 15.25 × 10^3^ cells/well. After 24 h growth at 37 °C in a humidified atmosphere (Day 0), the culture medium with the analyzed complexes (TY-Ag and BSA-TY-Ag) was added to the cells. The concentrations of TY of 40 µM and 20 µM were used for the preparation of the complexes with Ag^+^ (in the molar ratio TY-Ag = 1:0.5) and additionally with albumin (in the molar ratio BSA-TY = 1:10). The incubation was continued for the next 24, 48, and 72 h at 37 °C in a humidified atmosphere. The MTS assay (Promega, Madison, WI, USA) was used to determine the influence of the Ag^+^ complexes (TY-Ag or BSA-TY-Ag) on the proliferation of T24 cells. The absorbance was measured at 570 nm using an Epoch Microplate Spectrophotometer (BioTek Instruments Inc, Winooski, VT, USA). Four replicate wells were used for each experiment. The effect of TY-Ag or BSA-TY-Ag complexes was presented as the proliferation curves [82,83].

- Cell Survival Analysis

Caspase-3 Activity Assay: Apoptosis was analyzed by evaluation of the Caspase-3 activity. Briefly, cells were seeded and cultured on 24-well plates with TY-Ag in 4 concentrations of Ag^+^: 3.75 µM, 7.5 µM, 15 µM, and 30 µM complexed with TY (60 µM) for 24 and 48 h, then the cells were lysed in lysis buffer 0.05 M Tris/HCl, pH 7.6, 0.1 M NaCl, 0.001 M EDTA, 1% Triton X-100, and assayed for activity of Caspase-3 (DEVD-like caspase activity). An identical experiment was performed in parallel to estimate the cell number in each well (MTS). The amount of aminofluorocoumarin (AFC) released during incubation with Ac-DEVD-AFC was measured using a spectrofluorometer. For clarity of representation, fluorescent signal reflecting Caspase-3 activity was normalized to cell number (DEVD-like caspase activity/MTS cell number) and expressed as a fold increase in relation to untreated/control sample. The results show a single experiment performed in triplicates. The obtained data were averaged, and the standard deviation is represented in graphs by bars [84].

Flow cytometry: Apoptosis was analyzed with PE Annexin V Apoptosis Detection Kit I (BD Biosciences, San Jose, CA), following the manufacturer’s instructions. Briefly, T24 cells (3 × 10^5^) were seeded and cultured overnight on a six-well plate with TY and TY-Ag for 24 h. Approximately 1 × 10^5^ cells were stained with Annexin V for 15 min at RT in the dark. After the incubation, 5μL of propidium iodide and 400 μL of buffer solution were added prior to the cytometric analysis alone. Fluorescence intensities of treated samples and controls were analyzed by flow cytometry on a BD FACSCantoTM (Becton Dickinson, New York, NY, USA). Experiments were performed at least twice.

- Scratch Assay

Confluent T24 cells were cultured with McCoy medium with 0.5% BSA for 24 h. Next, cells were treated with TY-Ag for 24 h. The control cells were cultured under identical conditions without TY and TY-Ag (non-treated cells). The monolayer was scratched with a pipette tip and washed with phosphate-buffered saline (PBS) to remove floating cells. The scrape was monitored and photographed after 24 h incubation. The pictures were analyzed using Image J software (National Institute of Health, Bethesda, MD, USA), as described previously [85].

- Cell Cycle Assay

Cell cycle analysis was performed with the PI/RNase Staining Buffer assay (Becton Dickinson, New York, NY, USA). In this method, PI enters the cell through damaged membranes and can integrate into double-stranded DNA producing a fluorescent signal when properly excited. PI can also integrate into double-stranded RNA, therefore cells are treated with RNAse (RNA digestion). The amount of PI attached to the cell correlates with the amount of DNA, and thus allowed for the quantification of cells at particular phases of the cell cycle. For this purpose, cells, after 48 h of incubation, were rinsed with PBS and then fixed with cold 70% ethanol for 2 h. The cells were then washed again with PBS and resuspended in staining buffer (PBS with 100 µg/mL RNase A, 50 µg/mL Propidium Iodide) for 15 min in the dark. Acquire data on a flow cytometer (Becton Dickinson, Canto, New York, NY, USA). The analysis of the cell cycle was carried out in the ModFit program.

## 5. Conclusions

To conclude, our results suggest that TY-Ag and BSA-TY-Ag, also with CR, are promising complexes in antimicrobial therapy and in the treatment of human urinary bladder carcinoma during inflammation and cancer.

The complexes can be effective in the removal of the bacterial film. TY-Ag or BSA-TY-Ag are new potential drugs and may become important therapeutic options in human urinary bladder carcinoma treatment or/and important antimicrobial agents as an alternative to antibiotics. Treatment of T24 cells with such inhibitors diminishes proliferation and migration of T24 cells and induces apoptosis. The ability to bind TY-Ag to BSA will protect the drug from uncontrolled release and ensure targeted delivery directly to the desired site when such a system is intravenously administered. Binding to CR will increase the solubility of the system as well as enable targeted transport using antigen-antibody systems. Based on our findings, it can be concluded that the TY-Ag complex may be a potential cytotoxic agent against cancer cells and bacteria. Considering the potential of these complexes, they may be recommended for biomedical applications after further in vivo/clinical studies.

## Figures and Tables

**Table 1:** Concentration of TY and Ag^+^ in four samples obtained by the serial dilution (TY-Ag molar ratios was 1:1).

No.	TY [mM]	Ag^+^ [mM]
1.	7.5	7.5
2.	0.9	0.9
3.	0.1	0.1
3.	0.03	0.03

**Table 2:** Minimum Inhibitory Concentration (MIC) of silver in complexes with TY or with BSA-TY in different molar ratios for two reference strains: *E. coli* and *S. aureus* determined with agar diffusion method.

Antibacterial Agents	*E. coli* (EC, ATCC^®^ 35218^M^)	*S. aureus* (SA, ATTC^®^ 29213^TM^)
TY-Ag (1:0.5)	MIC _Ag+_: 0.019 mM	MIC _Ag+_: 0.019 mM
BSY-TY-Ag (TY-Ag = 1:0.5; BSA-TY = 1:10)	MIC _Ag+_: 0.019 mM	MIC _Ag+_: 0.039 mM

**Table 3:** Minimum Bactericidal Concentration (MBC) of silver complexes with TY or with BSA-TY in different molar ratios for two reference strains: *E. coli* and *S. aureus*.

Antibacterial Agents	*E. coli* (EC, ATCC^®^ 35218^TM^)	*S. aureus* (SA, ATTC^®^ 29213^TM^)
TY-Ag (1:0.5)	MBC _Ag+_: 0.036 mM	MBC _Ag+_: above 1.12 mM
BSY-TY-Ag (TY-Ag =1:0.5; BSA-TY = 1:10)	MBC _Ag+_: 0.07 mM	MBC _Ag+_: above 1.12 mM

**Table 4:** Concentration of silver, TY and BSA in complexes with TY or with BSA-TY.

Ag^+^ (mM)	TY (mM)	BSA (mM)
4.5	9	0.9
2.25	4.5	0.45
1.12	2.25	0.22
0.56	1.12	0.11
0.28	0.56	0.05
0.14	0.28	0.03
0.07	0.14	0.01
0.036	0.07	0.007

## Data Availability

The data presented in this stude are available on request from the corresponding author.
